# Supplementary material for: Content-rich biological network constructed by mining PubMed abstracts
Source: BMC Bioinformatics. 2004 Oct 8;5:147. doi: 10.1186/1471-2105-5-147 (PMC528731; doi:10.1186/1471-2105-5-147)
Supplement: Additional File 2 — The original results of the above study (non-essential files are deleted to keep the file size under the limit set by BMC bioinformatics). [file 1471-2105-5-147-S2.bz2 › chilibotAdditionalFile2/dip50/247ID8978688E810/html/CUT1_CUT2.html]

 


 **CUT1** and **CUT2** 
  
Found 46 abstracts in PubMed, retrieved 46.  
 

 What does Google say? 
 PDF only 
| .edu only 

---

**Interactive relationship** (e.g. stimulation, inhibition, etc)

**Neutral relationship**- High gene dosage of  **cut1**  also complements  **cut2**  and cut10 mutants.  Ref: 2203537 Cell, 1990
- **Cut1**  is loaded onto the spindle by binding to  **Cut2**  and promotes anaphase spindle movement upon  **Cut2**  proteolysis.  Ref: 9635190 Curr Biol, 1998
- A palindrome binding protein PBP previously cloned binds only to palindrome 1 of  **cut1**  promoter but not palindrome 1 of  **cut2**  3 which contains two base substitutions.  Ref: 11756444 J Biol Chem, 2002
- We show here that the phenotype of ts  **cut2**  364 is highly similar to that of  **cut1** .  Ref: 8978688 EMBO J, 1996
- **Cut2**  is required for loading  **Cut1**  onto the spindle at prophase and  **Cut2**  proteolysis is needed for the active participation of  **Cut1**  in sister chromatid separation.  Ref: 9635190 Curr Biol, 1998
- The essential function of the  **Cut1**   **Cut2**  complex which ensures sister chromatid separation may be regulated by  **Cut2**  proteolysis.  Ref: 8978688 EMBO J, 1996
- The association between  **Cut2**  and  **Cut1**  is needed for the localization of  **Cut1**  to the spindles, as  **Cut1**  remains unbound to the spindle if complex formation is impaired.  Ref: 9635190 Curr Biol, 1998
- **Cut1**  protein concentrates along the short spindle in metaphase as does  **Cut2** .  Ref: 8978688 EMBO J, 1996
- Thus, CTF1 beta is involved in the constitutive expression of  **cut2**  that causes production of low levels of cutin monomers that strongly induce  **cut1**  using CTF1 alpha as the transcription factor.  Ref: 11756444 J Biol Chem, 2002
- Amino acid sequence deduced from the nucleotide sequence of  **cut1**  and  **cut2**  3 matched with that of the peptides from cutinase 1  [ **CUT1** ]  and cutinase  [ **CUT1** ]  2, respectively, isolated from F. solani pisi grown on cutin as the sole carbon source.  Ref: 11756444 J Biol Chem, 2002
- In yeast, CTF1 beta transactivates  **cut2**  promoter but not  **cut1**  promoter unless its palindrome 1 is mutated, unlike CTF1 alpha which transactivates  **cut1** .  Ref: 11756444 J Biol Chem, 2002

**Non-interactive relationship** (e.g. studied together, co-existance, homology, etc.)

- **Cut1**  approximately 200 kDa and  **Cut2**  42 kDa associate, as shown by immunoprecipitation, and co sediment as large complexes 30 and 40S in sucrose gradient centrifugation.  Ref: 8978688 EMBO J, 1996
- Single mutants in  **cut1**  or  **cut2**  did not effect sporulation.  Ref: 1934126 Curr Genet, 1991
- In this review, the roles of two proteins essential for the onset of anaphase in fission yeast,  **Cut2**  securin and  **Cut1**  separin  [ **CUT1** ] , are discussed with regard to cell cycle regulation, and compared with the postulated roles of homologous proteins in other organisms.  Ref: 10651900 Genes Cells, 2000
- the functions of the gene products of  **cut1**  and  **cut2**  are closely interrelated.  Ref: 8978688 EMBO J, 1996
- CONCLUSIONS Complex formation between  **Cut1**  and  **Cut2**  is needed for the onset of normal anaphase.  Ref: 9635190 Curr Biol, 1998
- Fission yeast  **Cut1**  and  **Cut2**  are essential for sister chromatid separation, concentrate along the metaphase spindle and form large complexes.  Ref: 8978688 EMBO J, 1996
- BACKGROUND The fission yeast Schizosaccharomyces pombe separase  **Cut1**  and securin  **Cut2**  are required for anaphase specific activation of proteolysis that leads to proper sister chromatid separation.  Ref: 12653962 Genes Cells, 2003
- Here, we have investigated the role of the  **Cut1**   **Cut2**  interaction in sister chromatid separation.  Ref: 9635190 Curr Biol, 1998
- Fission yeast  **Cut1**  separase resides in interphase cytoplasm and mobilizes to the spindle and the spindle pole bodies SPBs in mitosis, while  **Cut2**  securin remains in the nucleus from interphase to metaphase, and temporarily locates at the short spindle.  Ref: 12390246 Genes Cells, 2002
- ATP binding cassette ABC transporters mediating the uptake of carbohydrates comprise two subfamilies  **CUT1** ,  **CUT2**  that differ with respect to the chemical nature of their substrates, subunit composition, and conserved sequence motifs.  Ref: 11421277 Res Microbiol< MedlineTA>Res Microbiol, 2002
- We cloned three highly homologous cutinase  [ **CUT1** ]  genes,  **cut1** ,  **cut2** , and cut3, from Fusarium solani f.  Ref: 11756444 J Biol Chem, 2002
- RESULTS The carboxyl terminus of  **Cut2**  interacts with the amino terminus of  **Cut1** , and temperature sensitive  **Cut2**  mutants expressed  **Cut2**  proteins that contain substitutions in the carboxyl terminus and fail to interact with  **Cut1** , resulting in aberrant anaphase.  Ref: 9635190 Curr Biol, 1998
- **Cut2**  degrades during anaphase, but  **Cut1**  remains bound to the anaphase spindle.  Ref: 9635190 Curr Biol, 1998
- The  **cut1**  and  **cut2**  genes are essential for viability and interact genetically.  Ref: 8978688 EMBO J, 1996
- The  **cut1**  and  **cut2**  genes are cloned by transformation.  Ref: 2203537 Cell, 1990
- Mutations in the fission yeast  **cut1** ,  **cut2** , and cut10 genes uncouple normally coordinated mitotic events and deregulate, rather than arrest, mitosis.  Ref: 2203537 Cell, 1990
- BACKGROUND The  **Cut1**  and  **Cut2**  proteins of the fission yeast Schizosaccharomyces pombe form a complex and are required for the separation of sister chromatids during anaphase.  Ref: 9635190 Curr Biol, 1998
- roles of  **Cut1**  separin  [ **CUT1** ]  and  **Cut2**  securin.  Ref: 10651900 Genes Cells, 2000
- ... the double mutant  **cut1**   **cut2**  formed two spored asci.  Ref: 1934126 Curr Genet, 1991
